# Microcavity-assisted cloning (MAC) of hard-to-clone HepG2 cell lines: cloning made easy

**DOI:** 10.1186/s12896-024-00911-z

**Published:** 2024-10-15

**Authors:** Vid Mlakar, Laurence Lesne, Stefania Vossio, Isabelle Dupanloup, Yvonne Gloor, Dimitri Moreau, Marc Ansari

**Affiliations:** 1 CANSEARCH Research Platform for Pediatric Oncology and Hematology, Department of Pediatrics, Gynecology and Obstetrics, Faculty of Medicine, University of Geneva, Geneva, Switzerland; 2 University of Geneva, School of Chemistry and Biochemistry - Sciences II, ACCESS Geneva, Quai Ernest Ansermet 30, 1211 Geneva 4, Switzerland; 3 Swiss Institute of Bioinformatics, Lausanne, Switzerland; 4 Division of Pediatric Oncology and Hematology, Department of Women, Child and Adolescent, University Geneva Hospitals, Geneva, Switzerland

**Keywords:** Cloning, Single cell, Cell line, Microcavity, Hard-to-clone, Sorting, HepG2

## Abstract

Cloning is a key molecular biology procedure for obtaining a genetically homogenous population of organisms or cell lines. It requires the expansion of new cell populations starting from single genetically modified cells. Despite the technical progress, cloning of many cell lines remains difficult. Cloning often fails either due to the strenuous conditions associated with manipulating cells or because many cells don’t tolerate a single-cell state. Here we describe a new cloning method utilizing low adhesion microcavity plates. This new technique, named microcavity-assisted cloning (MAC) was developed to clone difficult-to-clone HepG2 cells. The clones were produced following CRISPR/Cas9 knockout of the GSTA1 gene by a random distribution of 200, 400, and 800 cells into 550 microcavities of a 24-well low adhesion plate originally designed for the culture of spheroids. The knockout of GSTA1 was verified at the protein level using Western blotting. The advantages of the MAC method are its low cost, ease of the procedure, and the possibility of scaling up the throughput and automatization.

**Supplementary Information:**

The online version contains supplementary material available at 10.1186/s12896-024-00911-z.

## Introduction

Cloning is a key molecular biology procedure for obtaining a genetically homogenous population of organisms or cell lines. The procedure is important in industry and research to obtain a uniform starting material either for production or experimental purposes. There are many different techniques to perform cellular cloning but the two most widely used ones to date rely on either fluorescent activated cell sorting (FACS) [1, 2] or limited dilutions [3]. Other alternatives include single-cell pipetting with microfluidic devices to distribute single cells into separate wells [4], cloning in Petri dishes using rings [5], isolating colonies in semi-solid agar [6, 7], or using specially designed custom-made chips [8, 9]**.** While FACS is by far the most efficient and widely used method, it has several drawbacks including the high cost of initial investment in the sorting apparatus, the need for highly qualified personnel, and the incapacity of some cell types to form a colony starting from a single cell fully isolated in a well of a titration plate [10]. Several solutions have been proposed to overcome the limitations associated with strenuous FACS sorting conditions. Most solutions are based on “gentle” manipulation of the cells using different microfluidic devices for single-cell manipulation [4, 8, 9] and on the use of conditioned media or feeder cells to help the growth of single cells [10]**.** However, these devices can be hard to produce or cumbersome to obtain. Moreover, they might not guarantee clonality as their adhesive surface allows cells to attach to the surface and migrate thus contaminating neighboring wells. In addition, they still fail to address the problem of single-cell colony formation. The limited dilution alternative is a cumbersome method with low production efficiency, and similar to techniques using rings or semi-solid agar, it is difficult to ensure that the population is derived from a single cell [3]. Due to the increasing demand for biotechnological products and stringent quality control standards of the industry [11] other methods like cell printing [12, 13] and complex microfluidic devices [14] with integrated optics have been developed. However, these devices have very high throughput, and their acquisition and running costs are justified only when large-scale cloning projects are performed. With the advent of CRISPR/Cas9 genome editing, facilitating genetic manipulation of cell lines, the need for efficient, high throughput but low-cost cloning method adapted to any type of cells is greater than ever. In this article, we describe an innovative microcavity-assisted cloning (MAC) method that addresses some of these needs.

## Methods

### Cell lines

HepG2 hepatoblastoma cells obtained from ATCC (USA) were kept in a DMEM/F12 medium (ref: 31331–028, Gibco, USA) supplemented with 10% FCS and 1% penicillin/streptomycin. A cell line with less than 10 passages was used for the cloning procedure. Before MAC HepG2 cells were passaged twice using extensive trypsinization to reduce cell clumps and to ensure even and single-cell growth of the cells.

### Distribution into microcavities and growth of spheroids

**Fig. 1:**
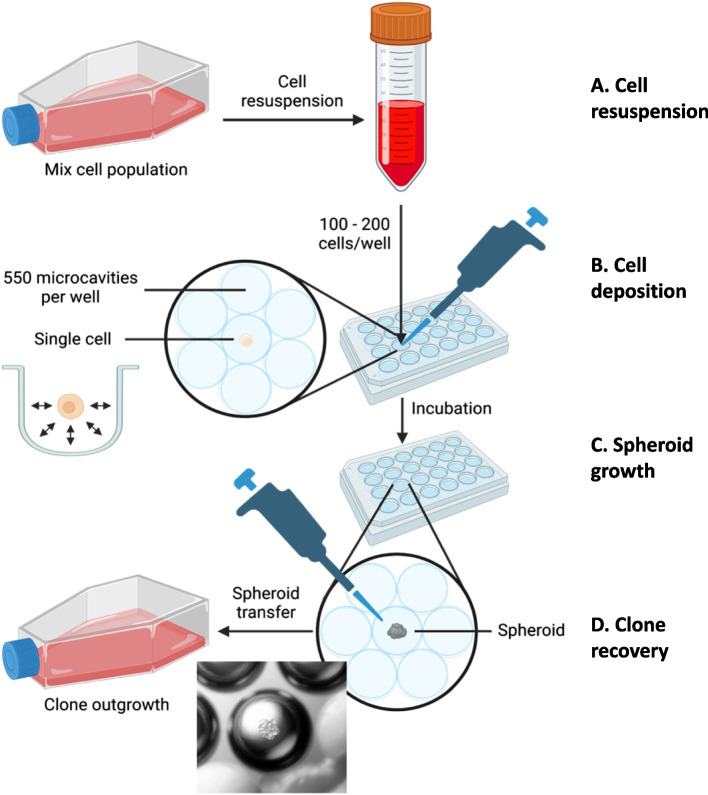
Workflow of the MAC cloning procedure. **A** Cells are resuspended. This step is critical, particularly in cell cultures that tend to aggregate. **B** 100 to 200 cells are deposited in each well containing 550 low-adhesion cavities. **C** Cells are incubated at 37⁰C for 10 days until the spheroids form. **D** Clone recovery. Using a standard laboratory pipet, spheroids can be transferred to a standard cell culture plate with the adhesive surface for outgrowth and testing

The core of the method is the use of a microcavity 24-well plate (Corning® Elplasia® 24-well Black/Clear Round Bottom Ultra-Low Attachment, Microcavity Plate, with Lid, Product number: 4441, Corning, USA) where each well contains 550 ultra-low attachment microcavities that prevent both attachment and migration of the cells from one cavity to the next (see Fig. 1 for procedure). The plates were primed using 500 µL of growth medium and centrifuged to remove air from the microcavities. Cells grown to 60 to 70% confluency were extensively trypsinized (5 min in 0.25% trypsin) and resuspended by vigorous pipetting to ensure disruption of the clumps and homogeneous cell suspension. Cells were counted using an automated cell counter Countess (Invitrogen, USA) and diluted to prepare suspensions containing 200, 400, 800, and 1600 cells/mL. 500 µL of each cell suspension was added to the wells of the primed plates so that wells contained 100, 200, 400, or 800 cells respectively in a final volume of 1 mL. Quadruplicates of each concentration were made. The plate was shaken for 5 min at room temperature and 100 rpm on a horizontal shaker to obtain a homogenous distribution of cells in the microcavities. After 15 min of incubation at 37⁰C, the microcavities were inspected for the presence of the cells using an Eclipse TS100 light microscope (Nikon, Japan). We scanned 50% (275 microcavities) of each well using an automated microscope (IXM-C, Molecular Devices, USA). The imaging was run in widefield mode (larger field depth) focusing at the bottom of the microcavity which allows for accurate detection of the cells in the microcavity. Although, phase contrast images allow a good resolution of doublets, cell distribution assessment was done using the Hoescht dye to ensure the proper distinction between singlets and doublets. Next, cells were incubated for 10 days at 37⁰C and 5% CO_2_. Half of the media was replaced with fresh media after 4 days of incubation. The growth of the HepG2 cells was monitored on days 1, 3, 4, 7, 8, and 10 after seeding.

### Transfer of the spheroids and harvest of proteins

After 10 days, cloned cells formed spheroids. Twenty were transferred to a conventional 24-well cell culture plate (Multiwell 24-well, Falcon, USA). The transfer (Fig. 1 – film) was done using a standard 10 µL laboratory pipet (Eppendorf, Germany) manually operated under a standard cell culture microscope Eclipse TS100 (Nikon, Japan) equipped with 5 × objective. An example of the procedure was filmed using an AxioCam MRc (Zeiss, Germany) camera mounted on an AXIO Vert.A1 light microscope (Zeiss, Germany). After the transfer, the colonies were grown for an additional 5 days before their treatment with 100 µL of 0.05% Trypsin and transferred to T25 culture flasks. Proteins were harvested from at least 2 million cells.

### CRISPR/Cas9 GSTA1 knock out in HepG2 cells

The plasmid pX458 was used to deliver all-in-one gRNA and Cas9 labeled with GFP in HepG2 cells. The gRNA sequence targeting GSTA1 was the following 5’-CTATGGGAAAGACATAAAGG-3'. Plasmid was delivered in HepG2 cells using Xtreme-gene HP transfection reagent according to the manufacturer’s instructions (Roche, Germany). The DNA to Xtreme-gene HP ratio was 1:2. Complexes were delivered on 200.000 cells in 6-well culture plates. After 3 days of incubation, GFP-positive cells were FACS sorted using MoFlo Astrios (Beckman Coulter, UK). Cells were expanded up to 2 million cells before cloning. Western blotting was used to detect the presence of the GSTA1 protein. Abcam: GSTA1 (ab180650) and β-actin (ab213262). Biorad: secondary goat anti-rabbit IgG (H + L) HRP antibody (170–6515).

### FACS cloning

HepG2 cells were cloned using MoFlo Astrios EQ cell sorter (Beckman Coulter). Before cloning, cells were trypsinized and suspended in the standard HepG2 growth medium described above. Dead cells were stained with Draq7 DNA intercalant dye (biostatus, ref DR70250, between 0.5um and 1 um per million cells). Singlet living cells were sorted into a 96-well plate containing 100 µL of conditioned medium. Cells were gated on morphology on FSC-A / SSC-A plot, Draq7 negative cells were selected, and doublets were excluded based on the SSC-W and FSC-W parameters. MoFlow Astrios was performing with 100 um nozzle size, and 20 psi sheath pressure. Cloning settings from Summit Software 6.3.1 were the following ones: abort mode: purify; Drop envelop: 0.5. Next, cells were incubated for 10 days at 37⁰C and 5% CO2 and then inspected visually at 5 × magnification for the outgrowth of clones.

### Statistics

The binomial distribution was used to estimate the expected number of microcavities with 1, 2, 3, 4, and more than 4 cells in each well.

$$p=\frac{N!}{n!\left(N-n\right)!}{(\frac{1}{M})}^{n}{(1-\frac{1}{M})}^{N-n}$$

We compared the expected and observed number of microcavities with the different numbers of cells in each well using a chi-squared test.

## Results

### Cell distribution in microcavities

**Fig. 2:**
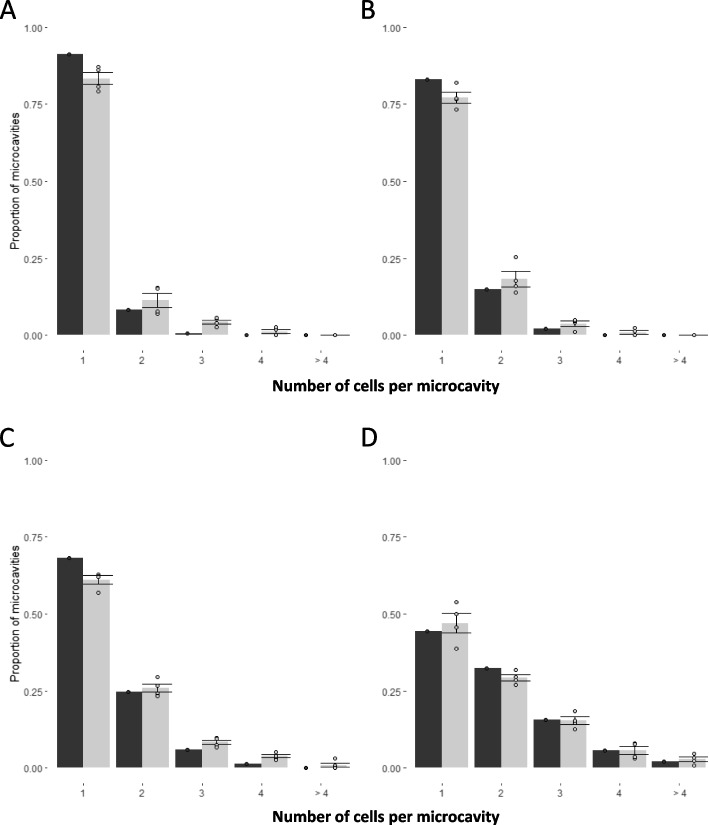
Distribution of cells in the microcavities of Elplasia 24-well microcavity plate after deposition of 100 (**A**), 200 (**B**), 400 (**C**), and 800 (**D**) cells per well. The expected distributions are shown in black and the observed distributions are in grey. The ideal ratio of one to multiple cells per well was obtained when depositing less than 200 cells per well. The error bars correspond to the mean plus or minus one standard deviation

Each Elplasia microcavity plate contains 550 microcavities per well (Fig. 1, Cell deposition). We first tested the optimal loading conditions of the cells in the microcavities. Following the probabilistic theory, cell distribution ought to follow a binomial distribution. This distribution was used to calculate the optimal number of cells to be deposited in a well. Based on our modeling results, we added either 100, 200, 400 and 800 cells per well. As predicted the best number of cells for obtaining a single cell per cavity in the total of 550 microcavities was between 100 and 200 cells. Each had approximately 80% of microcavities with single cells and less than 15% and 20% of microcavities with more than 1 cell, respectively (Fig. 2). Supplementary Fig. 1 shows the same binomial distribution but with the addition of empty microcavities. There was no significant difference between the expected and observed distributions (Fig. 2A: *p* = 0.441, 2B: *p* = 0.602, 2C: *p* = 0.475, 2D: *p* = 0.136).

### Spheroid development

**Fig. 3:**
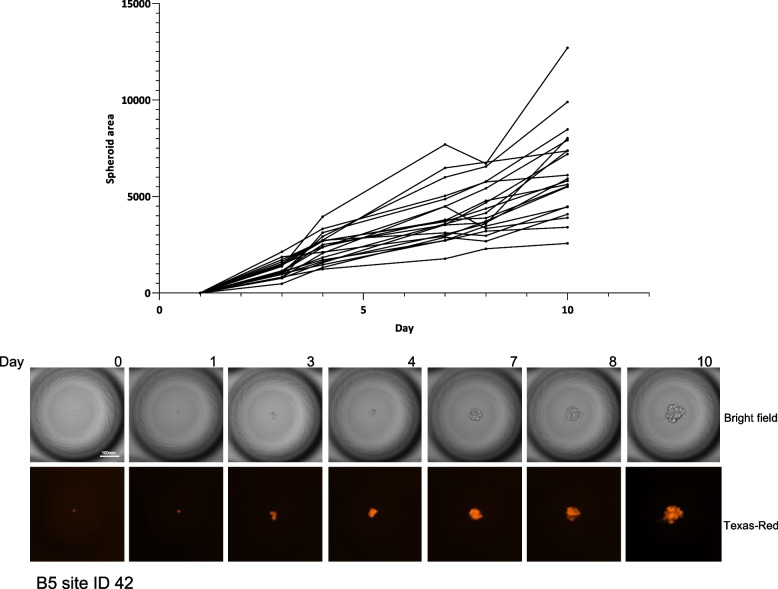
**A** Spheroid growth was monitored for 20 clones by measuring the clone surface area on indicated days. **B** Images show the growth of a spheroid through time

Over the next 10 days, we monitored the ability of single cells to grow and form spheroids inside the microcavities (Fig. 3, Spheroid growth). We concentrated on wells where we deposited 100 cells since more than 85% of the spheroids in those wells should be formed starting from a single cell and less than 15% of spheroids in this setup should be derived from more than one cell per microcavity. The outgrowth ratio (ratio between fully developed spheroid and positive well at the time of cell deposition) was 0.35 for HepG2 cells. Using single-cell FACS cloning into a 96-well plate all cells failed to grow. We observed a linear increase in the spheroid area over the next 10 days (*N* = 20) (Fig. 3).

### Efficiency assessment of clone isolation

**Fig. 4:**
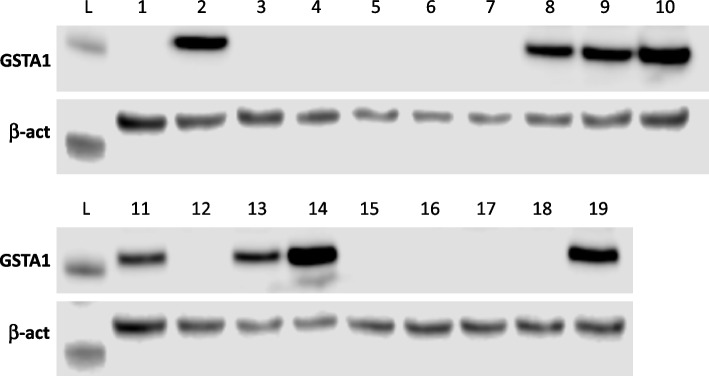
Proof-of-concept of the MAC method for CRISPR/Cas9 ko mutants. Western blot to detect GSTA1 in 19 clones obtained with MAC method. Out of 19 clones screened, 11 were GSTA1 knockouts confirming the ability of the method to isolate clonal cell populations. L = ladder (25 kDa)

To test the method's reliability in the very simple lab setup we cloned HepG2 cells treated with CRISPR/Cas9 system to make *GSTA1* knock out. To obtain the optimal number of spheroids from a single cell we deposited 200 cells per well and let spheroids develop for 10 days. 20 spheroids were manually picked under a microscope equipped with a 5 × objective, using a standard 10 mL laboratory pipet with transparent tips (Fig. 1, Spheroid transfer film) and transferred in a 24-well format plate for further outgrowth. Spheroids were not controlled for their outgrowth from a single cell. The WB showed that out of 19 spheroids, 11 were GSTA1 knockouts (Fig. 4). Only one spheroid failed to grow after the transfer.

## Discussion

Here we present an easy and efficient method for the production of clones using a commercially available ultra-low attachment microcavity 24-well plate in a standard P1 cell culture laboratory with standard equipment. The MAC was used to isolate *GSTA1* ko starting from a hard-to-clone HepG2 cell line. The key feature of this procedure is the use of 24-well plates lined with low-attachment surface microcavities. Low-attachment surfaces keep cells confined in the microcavities [15] by preventing the attachment to the plastic surface, thus the system is designed to avoid cross-microcavity cell movement. In addition, a forced suspension could maximize cell–cell interactions and might improve their colony-forming capacity [16]. The critical step in the execution of the MAC method is the removal of cell aggregates and homogenization of single-cell suspension. For this reason, the HepG2 cell line was passed one day before cloning to minimize cell aggregation. Alternatively, cells could also be passed through filters.

In the case of homogenous cell suspension, the cells are distributed into the microcavities according to a binomial distribution. Based on the distribution the best cavity-to-cell ratio is between 170 to 200 cells per 550 microcavities which should result in about 160 (> 80%) single-cell spheroids with only around 10 (< 20%) spheroids derived from two or more cells. However, in our experiment, the best ratio between single- and multiple-cell microcavities was obtained after seeding 100 cells per well. A slightly higher percentage of more than one cell per well as predicted by binomial distribution is probably due to the presence of cell doublets. This further stresses the importance of ensuring complete cell homogenization. Nevertheless, after seeding with 100 cells per well, we obtained a very high percentage (85%) of spheroids arising from a single cell and only 15% of spheroids arising from more than 1 initial cell.

After the plate preparation, the HepG2 spheroids needed about 7 to 10 days to grow enough to be ready for transfer into the larger culture format. The transfer of the spheroids was achieved manually using a conventional 10 µL tip pipet operated under a standard cell culture microscope with 5 × objective (Fig. 1). 20 clones were transferred into a standard 24-well plate in approximately 30 min. Colonies were left to grow in standard flat well cell culture plates for an additional 7 to 10 days before transferring them to the T25 flask. The result of the Western blot shows successful cloning of cells through obtaining pure cell populations showing no expression of the GSTA1 gene.

Assurance of clonality is an important issue in biotechnological production [11]. In addition to FACS and limited dilution, many other methods utilizing expensive equipment have been developed [2, 12–14]. In this paper we demonstrated the ability of MAC to produce clones and that the process could be visually followed at every step enabling the transfer of spheroids derived from a single cell. The limitation of the current publication is that we have not tested the actual probability of monoclonality. However, the technical possibilities to follow clone development from single cell to spheroid transfer were demonstrated. Therefore there is no reason to believe the process could not be made to meet important industrial standards.

In conclusion, after trying several different techniques, FACS, cloning rings, limited dilution, and single-cell pipetting, we developed our technique to successfully clone HepG2 cells. MAC is a rapid, reliable, and low-cost method for the isolation of cell clones that is easy to implement in a conventional cell culture laboratory. Importantly, it worked well with hard-to-clone cells that do not tolerate FACS nor form a colony from a single cell in conventional cell culture plates. The growth of cells could potentially be further improved by the use of a conditioned medium. Finally, the process could be both scaled up and automated by clone-picking machines, while using microscopy and image processing to ensure clonality of the obtained cell populations, a prerequisite for industrial processes.

## Supplementary Information

Supplementary Material 1.

Supplementary Material 2.

## Data Availability

No datasets were generated or analysed during the current study.
