# Supplementary material for: Microcavity-assisted cloning (MAC) of hard-to-clone HepG2 cell lines: cloning made easy
Source: BMC Biotechnol. 2024 Oct 15;24:81. doi: 10.1186/s12896-024-00911-z (PMC11481743; doi:10.1186/s12896-024-00911-z)
Supplement: Supplementary file 1 — Supplementary Material 1. [file 12896_2024_911_MOESM1_ESM.pptx]

## Slide 1
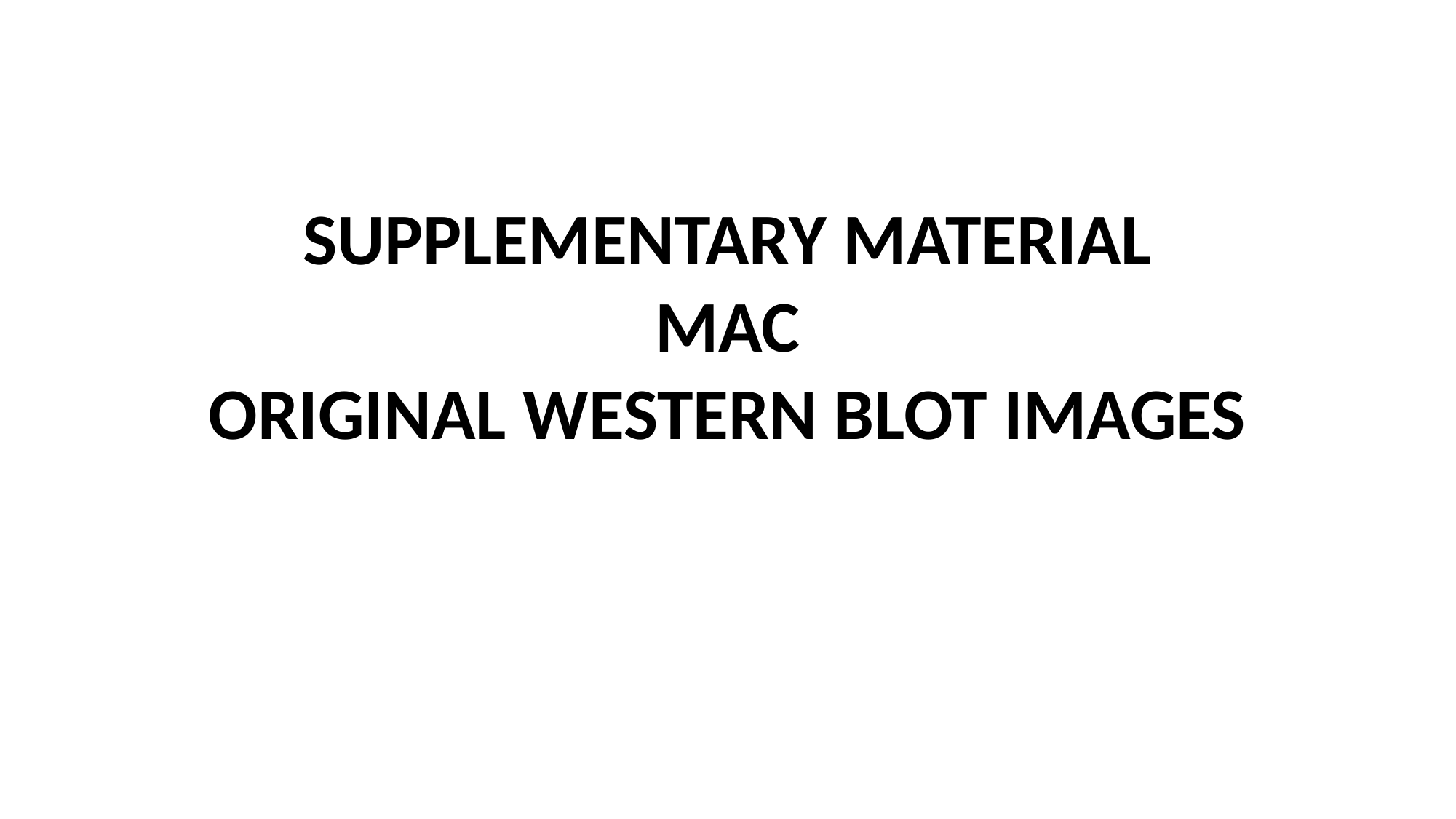

SUPPLEMENTARY MATERIAL
MAC
ORIGINAL WESTERN BLOT IMAGES

## Slide 2
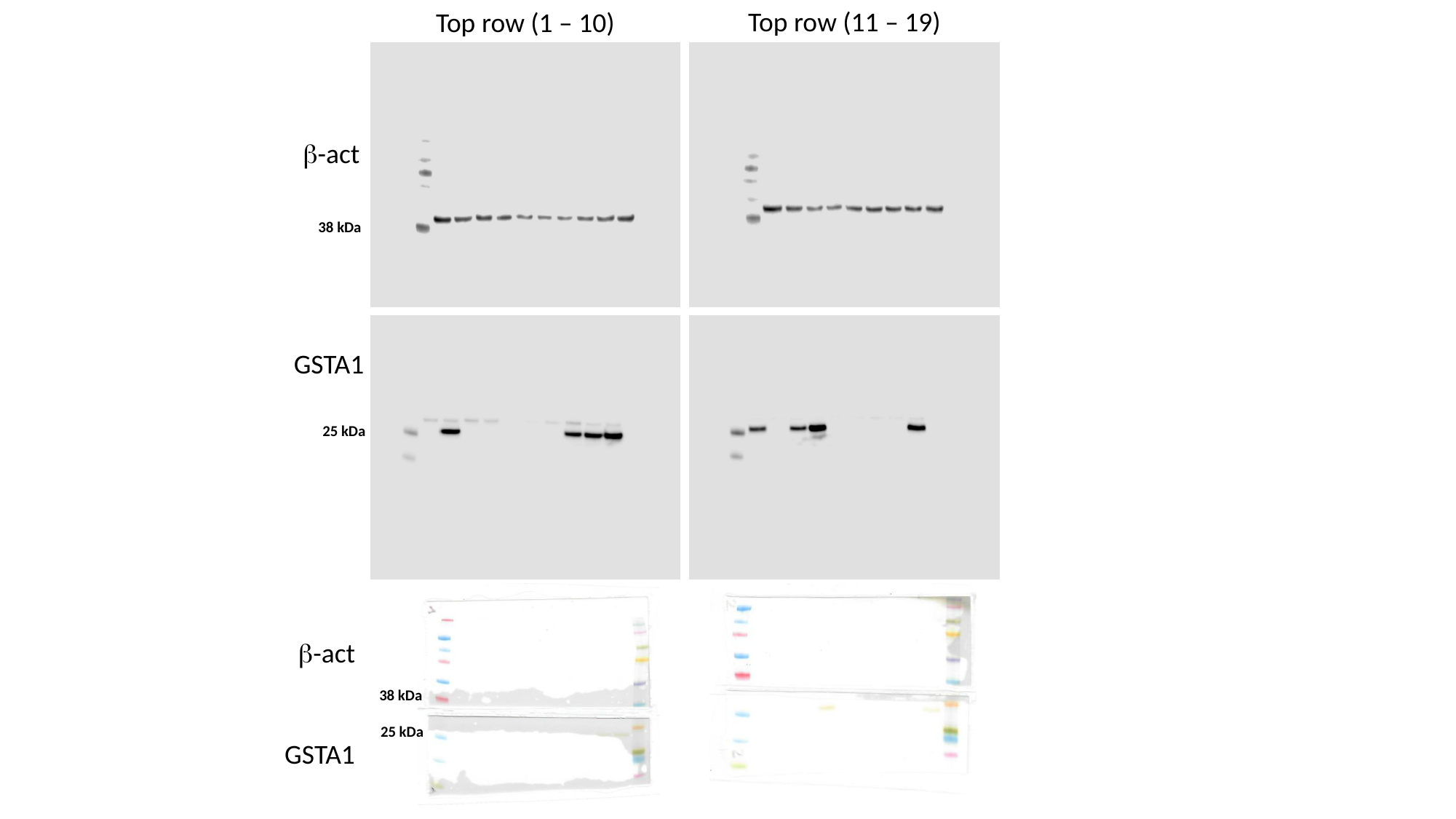

Top row (1 – 10)
Top row (11 – 19)
b-act
38 kDa
GSTA1
25 kDa
b-act
38 kDa
25 kDa
GSTA1
